# Inference of annealed protein fitness landscapes with AnnealDCA

**DOI:** 10.1371/journal.pcbi.1011812

**Published:** 2024-02-20

**Authors:** Luca Sesta, Andrea Pagnani, Jorge Fernandez-de-Cossio-Diaz, Guido Uguzzoni

**Affiliations:** 1 Department of Applied Science and Technology, Politecnico di Torino, Torino, Italy; 2 Italian Institute for Genomic Medicine, Torino, Italy; 3 INFN, Sezione di Torino, Torino, Italy; 4 Laboratory of Physics of the Ecole Normale Supérieure, CNRS UMR 8023 & PSL Research, Sorbonne Université, Paris, France; University of Wisconsin, Madison, UNITED STATES

## Abstract

The design of proteins with specific tasks is a major challenge in molecular biology with important diagnostic and therapeutic applications. High-throughput screening methods have been developed to systematically evaluate protein activity, but only a small fraction of possible protein variants can be tested using these techniques. Computational models that explore the sequence space *in-silico* to identify the fittest molecules for a given function are needed to overcome this limitation. In this article, we propose AnnealDCA, a machine-learning framework to learn the protein fitness landscape from sequencing data derived from a broad range of experiments that use selection and sequencing to quantify protein activity. We demonstrate the effectiveness of our method by applying it to antibody Rep-Seq data of immunized mice and screening experiments, assessing the quality of the fitness landscape reconstructions. Our method can be applied to several experimental cases where a population of protein variants undergoes various rounds of selection and sequencing, without relying on the computation of variants enrichment ratios, and thus can be used even in cases of disjoint sequence samples.

## Author summary

Advances in sequencing techniques have recently generated an explosion of protein sequence data. This represents an opportunity for scientists to develop theoretical and computational methods that can extract relevant biological information from these data samples. In this perspective, machine learning methods are proving to be particularly effective in the biological context. Since the majority of the accessible protein sequences are not-annotated, i.e. no information about the functional properties is known, unsupervised machine learning methods are particularly suited to tackle such raw sequence data. Here, we propose an unsupervised inference method which is meant to be applied to protein sequence data generated by an evolutionary process, whether it takes place in a controlled experimental framework or in-vivo. The method is devised to be simple enough to be applied to a plethora of different experimental setups, at the same time modeling the fundamental features of the dynamical processes underlying data generation. The ultimate goal of the method is to provide a sequence-fitness mapping that goes beyond the experimentally assessed sequence space, so to assign a quantitative functional score to each possible protein variant. The accurate knowledge of this mapping is key for several biological applications, such as biomolecule design and engineering, diagnostic and therapeutic treatments, and vaccine development.

This is a *PLOS Computational Biology* Methods paper.

## Introduction

The design of proteins to perform a given task (*e.g*. binding a target molecule) is a paramount challenge in molecular biology and has crucial diagnostic and therapeutic applications. Several high-throughput screening technologies have been developed to systematically assess protein activity. Despite the high parallelization of many techniques, a fundamental limitation lies in the small fraction of possible molecules that can be tested compared to the huge number of possible variants. Leveraging those data using effective computational models is crucial to overcome the obstacle by exploring in-silico the sequence space for the fittest molecules for a given function. We use the term *fitness* generically to refer to the protein activity under selection in a screening experiment (or during the *in-vivo* affinity maturation process). Several molecular activities can be selected in such experiments ranging from binding to a substrate to very complex phenotypes, such as conferring antibiotic resistance or multiple unknown interactions in a tissue.

Many machine-learning methods have been proposed recently to learn the protein fitness landscape from sequencing of high-throughput screening experiments [1–7]. Here, we propose a machine learning framework to target sequencing data derived from a broad class of experiments that use selection and sequencing to quantify the activity of protein variants. These experiments include, among others: *Deep Mutational Scanning* (DMS), where a library of protein mutants is screened in-vitro for different activities [8–21]; *Experimental Evolution* (EE), where a mutagenesis step adds diversity in the library after the rounds of selection [22–24]; sampling of the in-vivo immune response as in antibodies *Repertoire Sequencing* (Rep-Seq) [25]. Some of these experiments serve to select the fittest variants within the screened library while providing quantitative information about the protein activity landscape.

A basic quantitative measure of protein fitness can be obtained by computing the ratio between the relative frequencies of the variants in the populations before and after selection. This ratio, called *selectivity*, is a proxy for the probability that a variant survives the selection process, and has been widely used in the analysis of DMS experiments [8, 26]. Other approaches leverage more efficiently the same information, by parameterizing in some way the genotype-fitness map [7], or by developing adequate denoising procedures [27–29].

All these approaches evaluate the fitness from the temporal trajectory of variant abundances through the selection rounds. Conversely, many experimental setups are incompatible with the notion of a single variant trajectory in the population. Such is the case of EE, where a mutagenesis process occurs alongside selection that modifies the pool of individual variants from one round to the next [22, 23]. Depending on the interplay between mutational drift, selection strength, and the fitness landscape, the probability to re-sample previously seen variants can be very small after some rounds. Most variants do not persist through the whole time series and are often observed only once. In other setups, a severely undersampled regime precludes the repeated observation of individual variants. In repertoire sequencing, the coverage is generally too low in comparison to the large number of receptors present in an immune repertoire, which implies that individual sequences are not sampled more than once.

For other *in-vitro* screening experiments, factors such as the selection strength, the number of rounds, the shape of the fitness landscape, the size of the initial library, and sequencing coverage, can limit the ability to observe a relevant fraction of the possible variants. In these cases, we cannot detect the time trajectory of the frequency of most variants and thus we cannot compute an enrichment ratio. Nevertheless, it is still possible to make inferences about the fitness landscape. Another possible approach involves a dimensionality reduction of the protein sequence space through the modeling of the evolution of the distribution of the variants as selection proceeds.

Here, we propose AnnealDCA, a simple but effective strategy to perform protein fitness landscape inference, which can be applied to different experiments and types of data. Our approach is inspired by the simulated annealing method [30] from statistical physics to solve optimization problems. The different experimental rounds can be viewed as a cooling process, where an effective temperature is gradually reduced across successive rounds, and the selective pressure becomes increasingly dominant. The general mathematical framework and the associated statistical inference method can be applied to most of the experimental cases where a population of protein variants undergoes different rounds of selection, and a subset (or all) rounds are sequenced. Datasets of this type include, among others, protein screening experiments with one or multiple panning rounds, and the collection of Rep-Seq samples at different infection times.

To demonstrate the effectiveness of our scheme, we apply the method to antibody Rep-Seq data of immunized mice and we predict the antibody affinity towards its cognate antigen. We further test the method in more controlled experiments and assess the quality of the in-silico reconstructed fitness landscape.

## Method

To describe our method, we start for the sake of simplicity by considering a simple screening experiment of an initial library that takes place over several panning rounds. Other experimental setups will be described next. We define *P*_*t*_(**S**) as the probability of observing a sequence **S** at round *t*. Eventually, *P*_*t*_(**S**) is the quantity we want to estimate from the sequencing data. We introduce a sequence-dependent *survival factor* *Q*_*t*_(**S**). This quantity is a measure of the probability that sequence *S* survives between round *t* − 1 to *t*. Similarly to [1, 31, 32], we assume that this quantity takes the following exponential form:

$$\begin{array}{c} Q_{t}\left(\text{S}\right)\propto \text{exp}\;(-\alpha_{t}E\left(\text{S}\right)), \end{array}$$ (1)

with a time dependent factor *α*_*t*_, modeling the scale of the selective pressure acting at round *t*. The time-independent function *E*(**S**), associates a statistical *energy* to the protein sequence **S**. Thanks to Eq (1), we can then express *P*_*t*_(**S**) as:

$$\begin{array}{cc} P_{t}\left(\text{S}\right) & \propto Q_{t}\left(\text{S}\right)P_{t-1}\left(\text{S}\right)\\ & \propto P_{0}\left(\text{S}\right)\prod_{t^{\prime }=1}^{t}Q_{t^{\prime }}\left(\text{S}\right)\\ & \propto P_{0}\left(\text{S}\right)(e^{-E(\text{S})}{)}^{\sum_{t^{\prime }=1}^{t}\alpha_{t^{\prime }}} \end{array}$$ (2)

up to a normalization constant.

Using Eq (2), we can express *P*_*t*_(**S**) as a product of the initial configuration probability *P*_0_(**S**) and the factor *e*^−*E*(**S**)^, raised to the sum of the selective pressures of all rounds. We can redefine such a sum as:

$$\begin{array}{c} \beta_{t}=\sum_{t^{\prime }=1}^{t}\alpha_{t^{\prime }}. \end{array}$$ (3)

Eq (3) can be interpreted as a fictitious inverse temperature, accounting for the cumulative selective pressure up to round *t*. In the absence of mutations and if the experimental conditions are the same for all rounds, Fisher’s fundamental theorem of evolution states that *α*_*t*_ is a decreasing function of time [33]. Thanks to Eq (3), we can transform Eq (2) as follows:

$$\begin{array}{c} P_{t}\left(\text{S}\right)\propto e^{-\beta_{t}E\left(\text{S}\right)}P_{0}\left(\text{S}\right). \end{array}$$ (4)

The accumulated selection, quantified by the inverse temperature *β*_*t*_, tends to drive the mass of the distribution *P*_*t*_(**S**) towards the minima of *E*. This mental picture is reminiscent of the simulated annealing process studied in statistical mechanics and other areas [30].

At *t* = 0, *P*_0_(**S**) is the distribution of the variants in the initial library. Since this library is randomly generated, it is supposed to be unrelated to the selection process, and consequently to fitness. We can model the distribution of the initial variants by another similar energy function *G*(**S**):

$$\begin{array}{c} P_{0}\left(\text{S}\right)\propto e^{-G(\text{S})} \end{array}$$ (5)

so that Eq (4) takes the following form:

$$\begin{array}{c} P_{t}\left(\text{S}\right)=e^{-\beta_{t}E\left(\text{S}\right)-G\left(\text{S}\right)}/Z_{t}, \end{array}$$ (6)

where *Z*_*t*_ = ∑_{**S**}_ exp(−*β*_*t*_*E*(**S**) − *G*(**S**)) is a time-dependent normalization factor, and the sum runs over all possible sequences.

**Fig 1:**
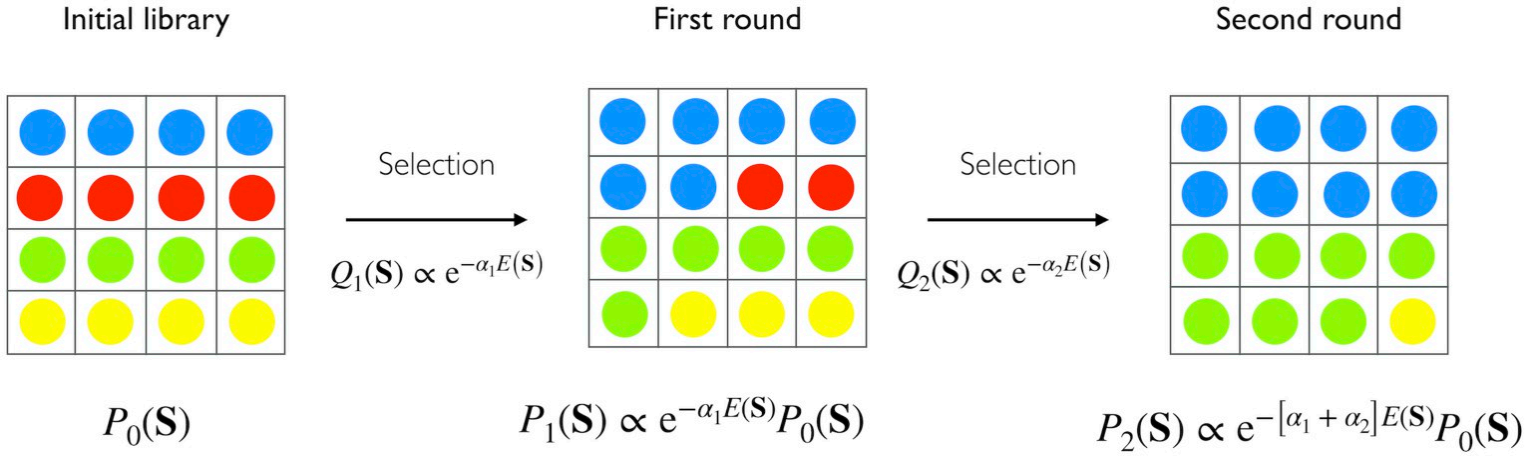
A simplified portrayal of the modeling of the selection process. Each color represents a different variant. Starting from the initial distribution of variants (which in this representation is uniform), the probability of observing a sequence in a subsequent round is shaped by the selection process, defined by the energy function *E*(**S**). *α*_*t*_ encodes the selective pressure at each transition. The arrows represent transitions between rounds, and underneath each round box, the related expressions of the model probability are reported.

Fig 1 shows a pictorial representation of the overall modeling of the experimental screening process. Notably, we do not need any explicit assumption on the specific temporal dependence of the inverse temperature, as the *β* factors can be inferred directly from the data.

### Fitness map

The genotype-to-fitness map here is encoded in the energy function *E*. The choice of its functional form and the related number of parameters to be inferred are eventually a trade-off between the expressive power and the actual availability of the sequence data to train the model. One of the simplest parameterizations is an independent site model, where each amino acid contributes additively to the energy:

$$\begin{array}{c} E\left(\text{S}\right)=-\sum_{i=1}^{L}h_{i}^{\left(E\right)}\left(\sigma_{i}\right) \end{array}$$ (7)

with parameters $h_{i}^{\left(E\right)}$ that depend on the identity of the amino acid *σ*_*i*_, present at position *i* along the sequence **S**.

A more complex parameterization is obtained by including pairwise epistatic interactions between all pairs of amino acids and is now widely used in structural biology [34, 35] and functional biology [36–40]. The resulting energy function takes the form of a generalized Potts model:

$$\begin{array}{c} E\left(\text{S}\right)=-\sum_{i=1}^{L}h_{i}^{\left(E\right)}\left(\sigma_{i}\right)-\sum_{i=1}^{L-1}\sum_{j=1+i}^{L}J_{ij}^{\left(E\right)}\left(\sigma_{i},\sigma_{j}\right). \end{array}$$ (8)

In comparison to the simple independent site model in Eq (7), the parameterization in Eq (8) is characterized by $O(L^{2})$ additional parameters, $J_{ij}^{\left(E\right)}$, to model the pairwise interactions. Furthermore, in cases where there is sufficient sequence variability, pairwise models have demonstrated the capacity to deliver superior performance when reconstructing fitness landscapes [37, 41].

### Model training

The model parameters are trained by maximizing the log-likelihood of the full dataset:

$$\begin{array}{cc} L(\theta ,\beta ) & =\sum_{t=\{\tau_{0},\ldots ,\tau_{f}\}}\sum_{m=1}^{M_{t}}w_{t}^{m}\;\text{log}\;P_{t}\left(S^{m}\right)\\ & =-\sum_{t=\{\tau_{0},\ldots ,\tau_{f}\}}\sum_{m=1}^{M_{t}}w_{t}^{m}(\beta_{t}E\left({\text{S}}^{m}\right)+G\left({\text{S}}^{m}\right)+\text{log}\;Z_{t}), \end{array}$$ (9)

where $w_{t}^{m}$ is the normalized abundance of the sequence *m* at time *t*, $w_{t}^{m}=N_{t}^{m}/\sum_{m^{\prime }=1}^{M_{t}}N_{t}^{m^{\prime }}$, {*τ*_0_, *τ*_1_, …, *τ*_*f*_} ⊂ {0, *t*_1_, …, *t*_*f*_} is the subset of sequenced rounds and ***θ*** = {***θ***^*E*^, ***θ***^*G*^} is the set of parameters of the energy functions.

The likelihood is the product of the probabilities to sample the observed sequences and frequencies from the model at each time point. It can be interpreted as minus the cross-entropy between the predicted distribution of the variants and the observed one, the empirical frequencies. The exact maximization of the likelihood involves the computation of the partition function of the model, whose computational complexity scales as $O(q^{L})$. To overcome this practical limitation, there are many approximate methods developed for specific parametrizations of the energy function. Other approaches, based on Monte-Carlo, are more general but might have convergence issues that are difficult to control in practice and are computationally costly. A very effective approach relies on the maximization of a quantity related to the likelihood, called the pseudo-likelihood function [42, 43], whose precise definition in the case of the Potts model (Eq (8)), is given in Section A of the S1 Text. A regularization term is added to the pseudo-likelihood to avoid overfitting. While Eq (9) is separately convex with respect to the energetic parameters and the inverse temperatures, this is no longer true when both are inferred simultaneously. To learn the parameters $\beta =(\beta_{\tau_{0}},\beta_{\tau_{1}},\ldots ,\beta_{\tau_{f}})$ it is possible to use an iterative optimization scheme: starting from an arbitrary set of ***β*** components, the energetic parameters ***θ*** are optimized. Next, the ***β*** components are updated while the ***θ*** are kept fixed. The two steps are iterated until both sets of parameters reach convergence. Some constraints can be imposed on the *β* parameters without affecting the expressivity of the model. In particular, it is possible to set $\beta_{\tau_{0}}=0$ and to fix a scale factor setting $\beta_{\tau_{1}}=1$.

### Methodological advancements and limitations

The fundamental motivation and distinguishing characteristic of the AnnealDCA method lies in its independence from the reliance on variant enrichment or, more broadly, the evolution of specific variant frequencies over time. This is in contrast to *Deterministic Rare Binding* (DRB) [7], a method previously introduced by some of the authors, that can process only variants present in at least two consecutive sampled rounds. Such a unique attribute empowers us to glean insights from experiments where the set of overlapping variants between rounds is notably limited. Such limitations might arise due to undersampling, the emergence of novel mutations, or other contributing factors. This capability is realized by implementing a dimensionality reduction technique that captures the temporal progression of the samples. In this context, we define dimensionality reduction as the ratio of the model’s number of parameters to the total number of possible variants. For a sequence of *L* = 100 amino acids, our model requires approximately *L*^2^ ⋅ 20^2^ + 20*L* ≃ 4 ⋅ 10^6^ parameters, while the number of possible sequences is 20^*L*^ ≃ 1.2 ⋅ 10^130^. This dimensionality reduction proves invaluable in effectively rectifying issues stemming from noise and undersampling. On the other hand, the AMaLa method (introduced in [6]) is explicitly designed to address experiments involving mutations. The energy function within the AMaLa framework was originally tailored to accommodate random mutations that reshape the population of variants throughout an experiment. This was achieved by incorporating an additional term derived from a generalized Jukes–Cantor model that describes the mutational step. These experiments typically start with a wild-type sequence and progress through a series of selection and mutation rounds. In the AnnealDCA approach, we do not explicitly model correlations between random mutations from one round to the next. In an undersampled regime, these correlations are expected to be weak and multiple rounds can be treated as independent samples. The approach shares similarities with various studies where a Potts model is inferred from a Multiple Sequence Alignment (MSA) of observed mutated viruses [44–48]. This inference is typically used to establish a prevalence landscape, often considered a surrogate for the intrinsic fitness landscape. However, it’s crucial to recognize that in the particular scenarios we investigate, the observed variant abundances (analogous to prevalence for viruses) are influenced by the stochastic composition of the initial library. It is important to note that the *G* part of the Hamiltonian serves the specific purpose of characterizing the bias introduced by the initial library and lacks a direct physical interpretation. Furthermore, *G* is not utilized in the subsequent analyses and validations. However, it effectively assimilates factors such as the impact of initially overexpressed variants and it remains crucial for accurately learning the energy component *E* related to the fitness. AnnealDCA’s applicability is subject to certain limitations, primarily due to the dataset statistics in terms of the size of the high-throughput screened library and the sequencing depth. Ensuring the accuracy of the probability model hinges on learning from sufficient statistics.

## Results

Most of the computational methods used to infer the fitness landscape from screening experiments rely on the computation of the enrichment/depletion ratios for a sufficiently large set of variants to train a regression model. The enrichment ratio is, in its simpler form, the ratio between the frequency of a variant at different rounds (see Eq (4) in the S1 Text). This quantity is a proxy for the ability of a variant to be selected during the process, namely, the fitness. However, several cases exist in which the temporal trajectory of the single variant is not detected. It can happen when: (i) the experiment is dominated by noise effects; (ii) the sequencing coverage is not adequate in comparison to the broadness of the library, and under-sampling effects might dominate; (iii) some mutations are introduced along the selection process at each round of the experiment. As a consequence, most of the variants sampled at different time points could be unique or in low copies, affecting the accuracy of the enrichment ratios estimate. Conversely, the generality of our approach makes it applicable to all the above cases. We demonstrate the efficacy and the versatility of the method by applying it to three different experimental setups, which are described briefly below. All references to the experiments and the datasets used are summarized in Table 1.

- *Antibody Repertoire Sequencing (Rep-Seq)*
  The Antibody Repertoire encompasses the diverse set of immunoglobulins present in an individual at a specific point in time. The Rep-Seq technique enables the study of a sample from this immunoglobulin repertoire. Our dataset was compiled from two sources: Khan et al. [49] and Gerard et al. [50]. In the former study, the authors sequenced IgG antibodies secreted by memory B cells and plasmablasts in non-immunized mice. In the latter, the same mouse clones were immunized against a specific antigen. Subsequently, the isolated IgG repertoire underwent high-throughput phenotypic assays using a microfluidic platform. This platform enriched the output in antigen binders, the authors estimates the final fraction of binders as 90%, as explained in detail in [50]. We can view the datasets as representing two distinct scenarios: the first as a sample before the immune response, and the second as a sample of clonally expanded antibodies responding to the antigen.
- *Deep mutational scanning (DMS)*
  These experiments combine high-throughput screening of a mutational library with sequencing to assess the effect of mutations on protein activity [51]. An initial library of protein variants undergoes one or multiple cycles of selection for a protein function (*e.g*. binding to a substrate). After a number of rounds, a sample of the variants is deep-sequenced to assess their abundances over time. Typical examples are *in-vitro* display experiments (*e.g*. phage display). The experiments and datasets we used in our study are described in Fowler et al. [26], Boyer et al. [52], and Wu et al. [53].
- *Experimental Evolution (EE)*
  EE follows a setup similar to DMS, with the difference that in this case, random mutations are repeatedly introduced before each panning round. In some cases, the experiment starts from a single wild-type protein. The experiment attempts to simulate *in-vitro* a natural Darwinian evolutionary process, where mutations explore the sequence space creating new genotypes whose phenotype is tested for the protein function. The experiments and datasets are described in the following two papers: Fantini et al. [22], Stiffler et al. [23].

**Table 1:** Experimental data overview.

Article	Experiment	Protein	# samples	# mutated residues	# variants
Khan et al. (2016) [49]	Rep-Seq	IgGHV	1	138*	1.9 × 10^4^
Gerard et al. (2020) [50]	Rep-Seq & sort	IgGHV	1 TT	138*	1340
Gerard et al. (2020) [50]	Rep-Seq & sort	IgGHV	1 GPI	138*	473
Boyer et al. (2016) [52]	DMS	Ab IgH	3 (round 1–3-6)	4	1.5 × 10^4^
Wu et al. (2016) [53]	DMS	GB1	2 (round 1–2)	4	1.0 × 10^5^
Fowler et al. (2010) [26]	DMS	WW	3 (round1–3-6)	25	9.8 × 10^4^
Fantini et al (2019) [22]	EE	TEM-1	3 (round 1–5-12)	286	2.6 × 10^5^
Stiffler et al. (2020) [23]	EE	PSE-1	2 (round 10–20)	266	6.15 × 10^5^
Stiffler et al (2020) [23]	EE	AAC6	3 (round 2–4-8)	148	1.6 × 10^6^

The table provides an overview of the experimental datasets employed to evaluate the method. It includes information such as the experimental configuration, the targeted protein, the quantity of available samples, the count of mutated residues, and the total number of variants generated during each experiment. In the Rep-Seq case, instead of the mutated part, is reported the aligned heavy chain sequence length(*). In our validation procedures, we compare distinct inference methods tailored to the respective experimental setups. For DMS, we compare with results obtained using the DRB method, In EE experiments, we utilize the AMaLa method, while for Rep-Seq experiments only the AnnealDCA method is available.

### Antibodies Repertoire Sequencing

We utilize our method on Rep-Seq data from antibodies to estimate the likelihood that a given antibody results from a specific immune response. Once the model is trained, it provides a parameterization of the probability function, which is then applied to design new antibodies. In essence, when the immune system responds to an antigen, the antibodies it produces should have a high affinity for that antigen. To achieve this, we work with two datasets, before and after the immune response: one from mice with unimmunized repertoires, referred to as the background or negative dataset, and another from mice with immunized repertoires (the positive set). In the case of the positive set, it is further enriched in binders through functional sorting using a microfluidic platform. Note that the latest experimental step increases the signal-to-noise ratio. However, in some instances, we may rely solely on samples from RepSeq data (see references [31, 32, 36]).

**Fig 2:**
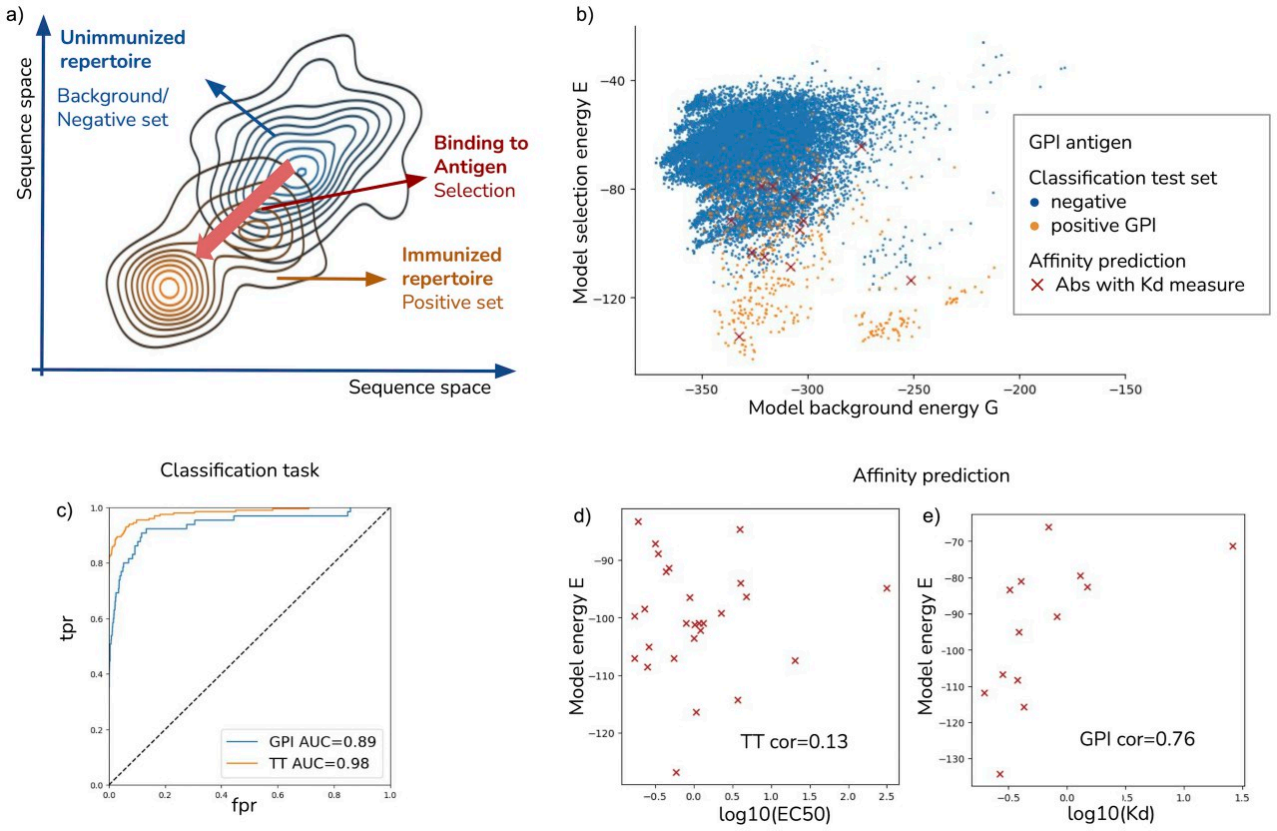
Method’s application to antibody Rep-seq data. (a) Depicts the inference of the selection process, where the initial antibody population represents the unimmunized repertoire (negative set). After the immune response, the library undergoes selection to bind the antigen (positive set), shaping the immunoglobulin population. (b) Displays a plot of background and selection energies for both negative and positive sets. Red crosses represent the test set of antibodies with affinity measures (panel e). The selection energy effectively distinguishes antibodies in the immunized repertoire from those in the unimmunized repertoire. (c) Classification Task: Demonstrates the model’s ability to discriminate between binders and non-binders by presenting results on a random test set composed of negative and positive antibodies. ROC curves for the GPI and TT cases yield area under the curve (AUC) values of 0.89 and 0.98, respectively. (d) and (e) Model energy vs. Experimental Affinity: Show scatter plots comparing the selection energy (y-axis in panel b) with affinity measures for a set of antibodies. Specifically, EC50 values for TT and *K*_*d*_ measures for GPI. Notably, a significant correlation (Spearman coefficient 0.76) is observed in the latter case. The GPI test set is indicated by red crosses in panel (b).

The fundamental concept is to model the probability of encountering an antibody in the positive set as the product of two probabilities: the background probability, which signifies the likelihood of finding an antibody in the negative set (unimmunized repertoire), and the selection factor. The selection factor describes the overall effective process of the immune response, including the impact of the enrichment platform. For a visual representation, please refer to Fig 2. The negative or background dataset contains sequences from the IgG heavy chain repertoire of three unimmunized BALB/c mice (the same type as for the positive dataset). The dataset is publicly available from the Observed Antibody Space [54] and the experimental setup is described in Khan et al. [49]. The negative dataset contains a total of 19772 unique IgG heavy chain sequences with the number of readouts. The positive datasets contain sequences of IgG heavy chain (VH) of immunized BALB/c mice repertoire sorted by a droplet microfluidics platform by the binding status of two immunogenic targets: Tetanus toxoid (TT) and Glucose-6-Phosphate Isomerase (GPI). The number of unique IgG heavy chain sequences in the two positive datasets is 3881 for TT and 3233 for GPI. All sequences were aligned using the Martin antibody numbering. The complete preprocessing pipeline is described in the Section B of the S1 Text.

We analyzed the similarity of positive and negative datasets in terms of different sequence statistics such as distances from consensus sequence, site conservation and covariance, alignment PCA, and germline distributions. These preliminary analyses however were not capable of revealing sensible differences between the two datasets (see more details in Section B and Fig A of the S1 Text).

We test the model for two distinct tasks: one is the classification of binders and not binders and the second is the model estimate of the binding affinity.

The first test assesses the ability of the model to discriminate between binders and not binders. For this purpose, we split the positive and background datasets into a training set (to learn the model) and a test set to validate the classification predictions. This validation procedure was chosen due to the lack of a large list of IgG labeled as binders of the two antigens (TT and GPI). Thus, we use the positive and background set as a proxy for binders/not binders labels. Fig 2 shows the results of the classification task. The sequences with low selection energy are likely to be part of the immune response to the specific antigen. The model can discriminate remarkably well the binders (of the positive set) and not binders (of the background set), as demonstrated by the ROC curve in the test sets of both targets (AUC 0.98 for TT and 0.89 for GPI).

In Gerard et al. [50], the authors reported the experimental measures of the dissociation constant *K*_*d*_ with GPI of a small set of antibodies (14 binders and 2 not-binders) and the EC50 values against TT for another small set (42 binders and 4 not-binders). Using this test set, we can test whether the inferred selection energy correlates with the antibody affinity or the neutralization power. As shown in Fig 2, (panel b), the antibodies in the test set (crosses in Fig 2, (panel b)) are evenly sampled from the sequence space from the positive ensemble and lay on the high-selectivity model energy region. The results show that inferred selection energy correlates with *K*_*d*_ GPI measures, while there is no significant correlation between selection energy and EC50 in the TT case (see Fig 2 panels d,e). Using our statistical model to quantitatively predict the activity of binding sequence variants in terms of binding affinity turns out to be a more challenging task, compared to the classification task. Although we do not have a clear-cut explanation for why we failed on the TT dataset (while doing a pretty decent job on the GPI dataset), we speculate that: (i) The activity measurements in the two experiments are different. For the GPI case, Surface Plasmonic Resonance (SPR) was used to establish the dissociation constant *K*_*d*_, while in the TT the EC50, i.e. the concentration required to obtain a 50% maximum antibody-ligand binding, was measured using ELISA. It is known that SPR measurements, albeit more complex, are generally more accurate compared to ELISA [55] because SPR measures the association *K*_*on*_ and dissociation rate *K*_*off*_ for the calculation of equilibrium dissociation constant (*K*_*d*_ = *K*_*off*_/*K*_*on*_), a more reliable measure for binding affinity. (ii) Although it is known that the immune response to TT is orchestrated by a complex interplay between the heavy and light chain [56], we could not take into account the contribution of antibodies’ light chains to neutralization, as the light chain of the background dataset was not sequenced. In other terms, if the contribution of the heavy chain alone seems to be sufficient to discriminate binder vs. not binder, it is possible that the contribution of both chains would be necessary for our model energy to better correlate with the binding affinity to TT.

### Deep mutational scanning (DMS)

Deep mutational scanning experiments are explicitly designed to quantify mutation effects on fitness. The broadness of the library and the sequencing depth are chosen to compute reliable enrichment measures for the variants ([8, 26]). Thus, approaches that leverage the enrichment ratios are more suitable to address these datasets. Nevertheless, it provides an interesting controlled case to assess the inference procedure and compare it to other tools. The screening experiment described in [26] probes the binding affinity of the human WW-domain with its peptide ligand. More than 6 × 10^5^ unique variants are generated in the initial library, which comprises almost all single point mutations, a fourth of the double mutations, and almost 2% of all three point mutations. Then, six rounds of phage display screening are performed, and rounds 3 and 6 are sequenced. In Boyer et al. [52], the library variability leans on a short sequence segment of the CDR3 region of an antibody’s heavy chain (*L* = 4). The library (chosen among 24 different scaffolds around the CDR3 region) is subsequently screened for three rounds of panning against a polyvinylpyrrolidone target. In the experiment described in Wu et al. [53], the variants library contains all possible mutations of four residues of the IgG-binding domain (GB1). The library is then screened to bind an immunoglobulin fragment target in a single round of selection.

**Fig 3:**
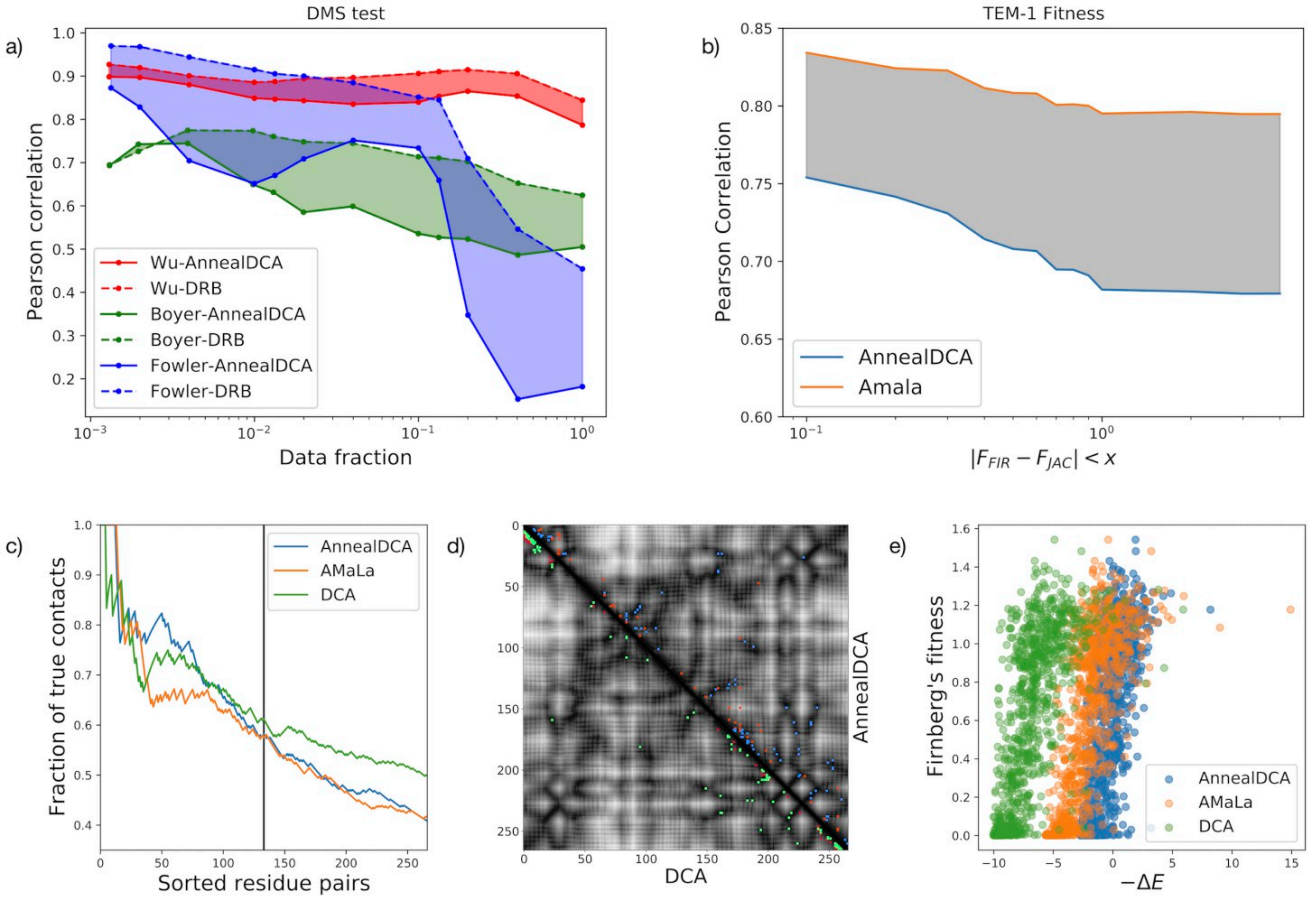
Comparative analysis on DMS and EE data. Panel (a) shows the overall performance of the method on DMS data: the Pearson correlation coefficient between inferred selective energies *E* and empirical log-selectivities (Eq (4) of the S1 Text). The correlations are reported as a function of data fraction pruned for the level of noise. The selectivity as a proxy for the fitness is more reliable for variants less affected by the noise, and consistently, for those variants the correlation with the energy is greater. Results are compared with the DRB method, which gains by using the enrichment information. Panel (b): comparison between AnnealDCA and AMaLa [6] for the reconstruction of the fitness landscape of TEM-1 from [22] data. Accuracy is quantified via the Pearson correlation between inferred energies *E* and independent fitness measurements [15, 16], as a function of the threshold discrepancy between the two datasets *x*. Panel (c): contact prediction sensitivity plot for the protein PSE-1. AnnealDCA (blue), AMaLa (green), and pseudo-likelihood DCA (orange) are inferred using data of [23] (DCA uses the last round only, as in [5]). On the y-axis, the *positive predicted value* is reported as a function of the first *L* residue pairs, sorted in decreasing order of the Frobenius norm (see Section A in the S1 Text). The vertical solid line coincides with *L*/2 residue pairs. Panel (d): Contact map related to panel (c). The plot is an *L* × *L* representation of the possible contacts between protein residues. The prediction of DCA (lower-left) and AnnealDCA (upper-right) are compared; correctly/incorrectly predicted contacts are respectively reported in green/red for DCA and blue/orange for AnnealDCA. Panel (e): scatter plot between selective energies inferred on [22] and fitness measurements of Firnberg et al [16] for a specific threshold value *x* = 0.8. Energies are rescaled with respect to the wild-type sequence Δ*E* = *E*(**S**) − *E*(**S**^*wt*^).

We perform a 5-fold cross-validation to test the inference method: for each dataset, a random selection of 4/5 trains the model while 1/5 operates as a benchmark. We compare the model energy function with the empirical log-selectivity, which is computed from the enrichment ratios and serves as a proxy for the variants’ fitness. The performance is then evaluated by the Pearson correlation between the model energies *E* and the log-selectivities in the test set. On all datasets, we observe high correlations as shown in panel (a) of Fig 3, where we report their trend with the number of sequences in the test set. Specifically, moving from right to left of the horizontal axis the sequences characterized by a higher uncertainty of the log-selectivity are progressively pruned. Several approaches can be employed in order to estimate selectivity uncertainties, ranging from bare Poisson counting, denoising procedures [28], or fit over different experiment replicas [29] (if available). Here we follow the approach outlined in [7], where the uncertainty is estimated as the error related to the regression procedure for estimating empirical selectivities *θ*^*m*^ (see Eq (4) in the S1 Text). Finally, we compare the results with the *Deterministic Rare Binding* (DRB) inference method developed in [7]. As expected, the DRB method performs better in all three datasets, as it uses the enrichment information. Nevertheless, we underline that we are still able to obtain an energy function highly correlated with selectivity, close to the best performance. Furthermore, for the Wu et al. dataset [53], we notice how the discrepancy between the two methods becomes very shallow, due to the broad coverage of sequence space. Lastly, we remark that DRB is unable to handle other datasets considered in this work.

### Experimental Evolution (EE)

Due to its flexibility, we can apply the method to experiments in which new protein variants appear via a mutagenesis step at each new round. In this case, as discussed in [6], we cannot compute the enrichment ratios and selectivity. The *G* Hamiltonian in Eq (6), although being time-independent, accounts for the mutational step in an effective manner, as is demonstrated by the high correlation between *G* and the Hamming distance from the wild-type sequence (see Fig I of the S1 Text). The *E* part, on the other hand, corresponds to the selection process.

We collect data from three experiments described in Fantini et al. and Stiffler et al. [22, 23]. The authors screen proteins responsible for antibiotic resistance in bacteria: TEM-1 and PSE-1 variants of the *β*-lactamase family and AAC6 protein of the acetyltransferase family. Starting from a wild-type protein, error-prone PCR creates new mutants at each round. Subsequently, the library undergoes a selection step in which bacteria equipped with the mutants are exposed to an antibiotic-rich environment. This cycle of mutagenesis and screening is repeated multiple times and for a subset of the panning rounds a sample of the library is sequenced. Specifically, 20 rounds of EE at an ampicillin concentration of 6*μ*g/mL are performed for PSE-1, among which rounds 10 and 20 are sequenced, whereas AAC6 mutants are subjected to 8 rounds at a kanamycin concentration of 10*μ*g/mL, of which rounds 2, 4 and 8 are sequenced. Finally, in [22] TEM-1 mutants are exposed to two different antibiotic concentrations: 25*μ*g/mL for all rounds but 5 and 12, for which the concentration is raised to 100*μ*g/mL. Out of the 12 experimental cycles, rounds 1, 5, and 12 are sequenced.

We performed two different validations to assess the inferred model:

(i) In the case of TEM-1 *β*-lactamase, we directly compare the model energy with independent fitness measurements related to antibiotic resistance, collected from [15, 16]. In [15] variants fitness is quantified in terms of *minimum inhibitory concentration* (MIC), that is, the minimum antibiotic concentration necessary to neutralize bacteria equipped with that variant. On the other hand, in [16], the authors directly measured the gene fitness (see Section A of the S1 Text). For our analysis, we mapped the measurements of [16] onto those of [15], following the procedure outlined in [37]. The results show that the inferred energy correlates with the experimental fitness (see panel (b) and (e) of Fig 3). The method described in [6], specifically designed for these experiments performs systematically better.
(ii) In the PSE-1 and AAC6 cases, for which fitness measurements are not available, we validate the model using the prediction of protein structure contact map as prescribed by the DCA method [34, 35]. Then, the predictions are compared to the crystallographic studies of the protein structures.

The contact predictions are obtained using the coupling parameters **J**, which quantify the interaction between residues in the DCA framework [34, 35]. We used the Frobenius norm of the *q* × *q* matrix *J*_*ij*_ to obtain a score quantifying the epistatic interactions between pairs of positions (see Eq (5) in S1 Text), on top of which we apply the *average product correction*. These residues are more likely to be found in spatial proximity in the folded structure as shown in panels (c), (d) of Fig 3.

## Discussion

Several machine-learning methods have been proposed for learning a protein fitness landscape using sequencing data obtained from high-throughput screening experiments [2, 7, 31, 32]. However, these methods require observation of the trajectory in multiple rounds of selection of a statistically relevant set of variants. This presents a limitation as detecting the single variants trajectory in the population is often unfeasible in many experimental setups. To overcome this issue, we propose AnnealDCA, a novel machine-learning framework inspired by the simulated annealing method from statistical physics [30]. This approach can handle sequencing data derived from a broad range of experiments that use selection and sequencing to quantify the activity of protein variants, including Deep Mutational Scanning, Experimental Evolution, and antibodies Repertoire Sequencing (Rep-Seq), among others.

In our approach, selection acts as a cooling process where the distribution of the population on the fitness landscape is gradually peaked around regions of higher fitness. The samples before and after the selection are considered at different statistical temperatures and the inference method decouples the distribution contribution due to the initial library and the time-dependent fitness part. The general mathematical framework and the inference method can be applied to most of the experimental cases where a population of protein variants undergoes a selective process and is sequenced at different times. Such datasets include, among others, protein screening experiments with one or multiple panning rounds, and the collection of Rep-Seq samples at different infection times.

To demonstrate the effectiveness of this approach, we applied the method to antibodies Rep-Seq data of immunized mice to predict the antibody’s affinity towards the antigen. We learned the model energies from the repertoire of mice unimmunized and subjected to two antigens. The model energy was then used to accurately classify binders and not-binders to the antigen. This was supported by the fact that it correlated well with experimental measures of the *K*_*d*_ antibody-antigen of a set of antibodies not used in the training of the model.

To further test our approach, we applied it to more controlled experimental setups using three Deep Mutational Scans experiments. The results of 5-fold cross-validation showed a high correlation between the inferred fitness landscape and the experimental selectivity. Additionally, we applied the method to Experimental Evolution experiments of three proteins responsible for antibiotic resistance in bacteria, where mutations are added to increase the variability and explore sequence space around a wild-type sequence. The model energy precisely described the antibiotic resistance measurements of a set of variants. Moreover, the model coupling parameters were able to predict the three-dimensional contact map with a level of precision comparable to other approaches.

In summary, AnnealDCA provides a simple but effective strategy that can be applied to different experiments and data types where a population of protein variants undergoes a selective process and is sequenced at different times.

## Supporting information

S1 Text Supporting information. Additional details about methods, datasets and further results.
(PDF)
