# Supplementary material for: Inference of annealed protein fitness landscapes with AnnealDCA
Source: PLoS Comput Biol. 2024 Feb 20;20(2):e1011812. doi: 10.1371/journal.pcbi.1011812 (PMC10878520; doi:10.1371/journal.pcbi.1011812)
Supplement: S1 Text — Additional details about methods, datasets and further results. (PDF) [file pcbi.1011812.s001.pdf]

Supplementary information for: Inference of annealed protein fitness landscapes with AnnealDCA

Luca Sesta,[1] Andrea Pagnani,[2,3] Jorge Fernandez-de-Cossio-Diaz,[4,5] and Guido Uguzzoni[2,5]

[1]*Department of Applied Science and Technology, Politecnico di Torino, Torino, Italy*

[2]*Italian Institute for Genomic Medicine, Torino, Italy*

[3]*INFN, Sezione di Torino, Torino, Italy*

[4]*Laboratory of Physics of the Ecole Normale Supérieure, CNRS UMR 8023 & PSL Research, Sorbonne Université, Paris, France*

[5]*Co-last author*

A. METHODS

Pseudo-likelihood

Direct optimization of the objective function in Eq (9) of the main text is infeasible due to the necessity to compute the partition function. To work around this limitation, we employed the so called *pseudo-likelihood* approximation [1, 2], which is based on single-site conditional probabilities. For the AnnealDCA model, such probabilities read:

$$P_t(\sigma_r|\boldsymbol{\sigma}_{\setminus r}) = \frac{\exp\left\{\beta_t\left[h_r^{(E)}(\sigma_r) + \sum_{i\neq r} J_{ri}^{(E)}(\sigma_r,\sigma_i) + h_r^{(G)}(\sigma_r) + \sum_{i\neq r} J_{ri}^{(G)}(\sigma_r,\sigma_i)\right]\right\}}{\sum_{a=1}^{q}\exp\left\{\beta_t\left[h_r^{(E)}(a) + \sum_{i\neq r} J_{ri}^{(E)}(a,\sigma_i) + h_r^{(G)}(a) + \sum_{i\neq r} J_{ri}^{(G)}(a,\sigma_i)\right]\right\}}. \quad (1)$$

The advantage brought about by Eq (1) is that the normalization factor can be computed as a sum over only q configurations. The objective function we ultimately optimize in order to infer AnnealDCA parameters becomes in this framework:

$$\begin{aligned} g\left(\boldsymbol{\theta}^E, \boldsymbol{\theta}^G, \boldsymbol{\beta}\right) &= -\sum_{r=1}^{L}\sum_{t=\{\tau_1,\ldots,\tau_f\}}\sum_{m=1}^{M_t} w_t^m \log P(\sigma_r = \sigma_r^m|\boldsymbol{\sigma}_{\setminus r} = \boldsymbol{\sigma}_{\setminus r}^m) \\ &+ \sum_{r=1}^{L} R_r^{(E)}\left(\mathbf{h}_r^{(E)}, \mathbf{J}_r^{(E)}\right) + \sum_{r=1}^{L} R_r^{(G)}\left(\mathbf{h}_r^{(G)}, \mathbf{J}_r^{(G)}\right) \\ &= \sum_{r=1}^{L} g_r\left(\boldsymbol{\theta}_r^E, \boldsymbol{\theta}_r^G, \boldsymbol{\beta}\right), \end{aligned} \quad (2)$$

where we introduced the regularization contribution, which we choose to be an l_2 penalty:

$$R_r\left(\mathbf{h}_r, \mathbf{J}_r\right) = \lambda_h\|\mathbf{h}_r\|_2^2 + \lambda_J\|\mathbf{J}_r\|_2^2 = \lambda_h\sum_{a=1}^{q} h_r^2(a) + \lambda_J\sum_{i\neq r}\sum_{a,b=1}^{q} J_{ri}^2(a,b), \quad (3)$$

depending on the auxiliary parameters λ_h and λ_J, which set the strength of the regularization. The l_2 regularization can be also intepreted as a Gaussian prior over the parameters in a Bayesian framework. Another significant advantage of the pseudo-likelihood approach is that the single site contributions g_r in Eq (2) can be optimized independently [2], and thus the parameters learning can be easily parallelized. The drawback of such asymmetric approach, is that it provides two different estimates for each coupling parameters J_{ij}: J_{ij}^i and J_{ji}^j, depending on whether the parameter was estimated from single site objective g_i

or g_j. The issue can be solved by combining together the two estimates, defining a unique coupling $J_{ij} = \left(J^i_{ij} + J^j_{ji}\right)/2$.

Selectivity

Enrichment ratios, which are defined as the number of copies of a specific sequence N^m_t at a certain time t divided by the number of copies at the previous time $N^{(m,t-1)}$ are often used to quantify fitness in protein screening experiments. From this, it possible to define the log-selectivity according to:

$$\log\left[\frac{N^m_t}{N^m_{t-1}}\right] = \theta^m + \alpha^m_t + \epsilon^m_t, \tag{4}$$

where α^m_t and ϵ^m_t are round dependent parameters respectively representing an amplification factor and a noise contribution (normally distributed). Thus, θ^m represents the log-selectivity of the sequence m, and it is estimated through a weighted least-squared regression, assuming that empirical counts are characterized by independent Poisson variance.

Contact prediction

In order to assess contacts prediction between protein residues, we use the Frobenius norm of the $q \times q$ matrix J_{ij} to obtain a score depending on pairs of positions:

$$F_{ij} = \|J_{ij}\|_2 = \sqrt{\sum_{a,b=1}^{q} J_{ij}(a,b)^2}. \tag{5}$$

The Frobenius score is subsequently modified according to the average product correction $F^{\mathrm{APC}}_{ij} = F_{ij} - F_{i\cdot}F_{\cdot j}/F_{\cdot\cdot}$, where the dots represent averages over the corresponding indeces. Residue pairs characterized by a higher value of F_{ij} have higher epistatic interaction and are more likely to be found in spatial proximity in the folded structure, as showed in panel (c)(d) of Fig 3 in the main text. Specifically, a pair of residues is considered to be in contact if at least two heavy atoms within the residues have a distance less than $8\mathring{A}$. Moreover, we restrict ourselves to consider pairs of residues i and j such that $|i-j| > 4$.

TEM-1 fitness measurements

In Section Results of the main text we tested the capability of AnnealDCA to infer the fitness landscape of TEM-1 β-lactamase from Experimental Evolution data [3]. In order to assess the meaningfulness of the inferred landscape we relied on the experimental fitness measurements realized in [4]. Since TEM-1 is the protein providing bacteria with antibiotics resistance, the mutants fitness will be quantified by a phenotypic trait related to the response to the antibiotics. Specifically, in [4] the authors define the fitness of a TEM-1 mutant as a weighted average of antibiotic concentrations:

$$f_m = \frac{\sum_{p=1}^{13} c_p^m \log_2(a_p)}{\sum_{p=1}^{13} c_p^m}, \tag{6}$$

where p is the index running over a collection of plates characterized by a different antibiotics concentration a_p, coinciding in the present case with ampicillin. The weights of the average are provided by c_p^m, that corresponds to the number of copies of mutant m in the p-th plate. Specifically, Eq (6) provides the unnormalized protein fitness, whose normalized version is given by $\phi_m = 2^{f_m}/2^{f_{wt}}$, where f_{wt} is the fitness of the wild-type sequence.

B. DATASETS

On GitHub at `https://gitlab.com/luca.sesta/AnnealDCA.jl` are available all the data used in this study and the codes to obtain them, that includes the repository of the AnnealDCA method to train the models, the Jupyter notebooks to reproduce the results and the script for the data preprocessing.

Antibodies Repertoire Sequencing

The data are described in Gerard et al. [5] and T.A.Khan et al. [6], the datasets publicly available and can be downloaded from `https://static-content.springer.com/esm/art%3A10.1038%2Fs41587-020-0466-7/MediaObjects/41587_2020_466_MOESM5_ESM.xlsx` and `http://www.ncbi.nlm.nih.gov/bioproject/311999` in the Raw FASTQ data format.

Once translated in fasta format (conversion script available on github repository) the sequences were align to martin numbering using ANARCI tool `https://github.com/oxpig/`

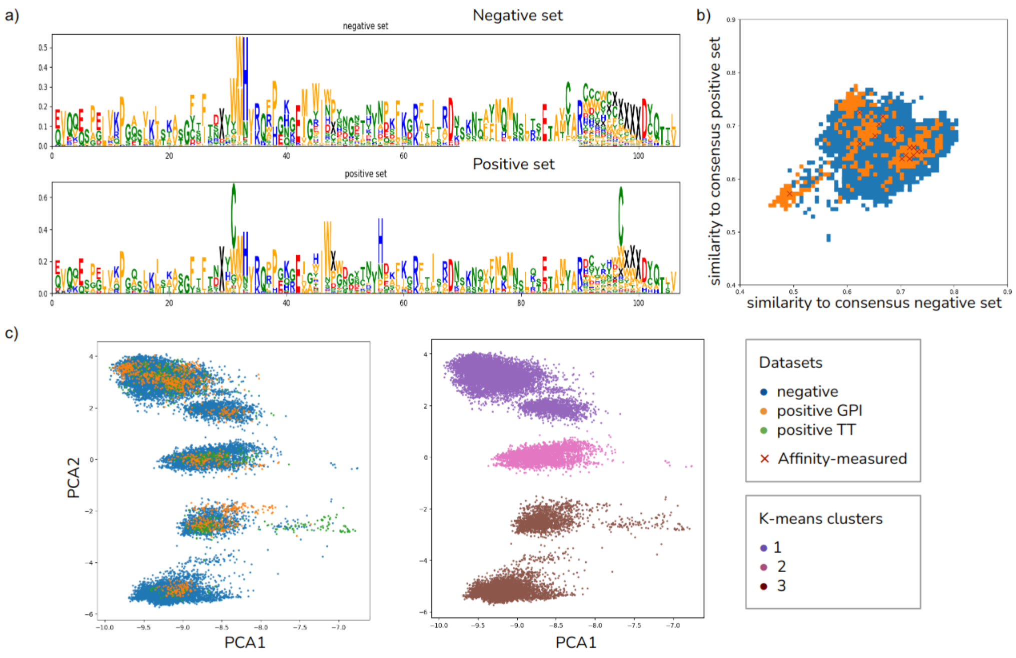

FIG. A. Antibodies repertoire sequencing data. In this figure three different ways to compare the background or negative sequence set (Khan et al.) and the positive set (Gerard et al. GPI antigen) are shown. In panel (a) the sequence logos representation of the two datasets, showing an overall similarity. In panel (b) is plotted the distance of each sequence in both datasets (negative are blue dots and positive orange dots) from the negative consensus sequence (x-axis) and positive consensus sequence (y-axis). In panel (c) the multi-sequencing alignment (MSA) of the negative dataset is used to compute eigenvectors. The sequences in the two datasets are then projected onto the first two components. On the right the same plot coloring the clusters obtained with K-means algorithm with k=3.

`ANARCI` [7]. Then, we filter out the aligned positions with more than 98% gap or 99% conserved residues. The full preprocessing piepline are available on Github.

In Fig A, the difference of the positive and negative datasets in terms of hamming distances from consensus sequence and the projections over the two first principal components are depicted, showing the overall high similarity of the positive and negative datasets.

Deep Mutational Scanning

The genome sequence data of Wu et al. [8] were downloaded from `https://www.ebi.ac.uk/ena/data/view/SRX958008`. For each DNA sequence a quality score and a pair of forward and backward reads are available. Low quality reads are discarded from the dataset,

and unique sequences are constructed from the forward and backward reads in such a way that, if a mismatch between two sites is present, the nucleotide with the higher quality score is retained. A similar procedure is followed for the data of Fowler et al. [9], whose raw genome sequences are downloaded from `https://www.ncbi.nlm.nih.gov/sra/SRA020603`. In [10], the authors study the binding capability of antibodies onto two targets, namely a short 9 nucleotides DNA segment and synthetic polymer named polyvinylpyrrolidone (PVP). Sequence variability is introduced in the short CDR3 segment, whereas different V_H scaffolds are used to define 24 different libraries. For our analysis, we focused on library F3, in which binding onto PVP is probed. Such data were downloaded from `https://www.pnas.org/doi/suppl/10.1073/pnas.1517813113/suppl_file/`.

For all DMS datasets, genome sequences are subsequently converted to protein sequences, from which the input data of the method are constructed. Specifically, the aligned unique sequences are concatenated to build up an $L \times M$ alignment matrix Z, with M the number of unique sequences and L the length of the proteins. Associated to this, an $M \times T$ matrix of counts is constructed, with T the number of available sequenced rounds of the experiment (usually including the initial library). In doing so, the sequences characterized by a high uncertainty value on the log-selectivity (Eq (4)) are filtered out.

Experimental Evolution

The Experimental Evolution data considered in this work are described in [3, 11]. Raw data related to [3] are available at the National Centre for Biotechnology Information Sequence Read Archive (SRA), therein accessible via the code PRJNA528665 (`http://www.ncbi.nlm.nih.gov/sra/PRJNA528665`). More refined data can be found at BioSNS site: `http://laborator-iobiologia.sns.it/supplementary-mbe-2019/`. Raw data related to [11] can also be found in the SRA, with accession code PRJNA578762, whereas refined data can be downloaded from `https://github.com/sanderlab/3Dseq`.

Similarly to what we did for the DMS data, the DNA sequences are firstly translated to amino acid ones. From these, an $L \times M$ alignment matrix Z and an $M \times T$ counts matrix are defined. Here, M coincides with the total number of unique sequences produced during the whole experiment, and T corresponds to the number of sequenced rounds.

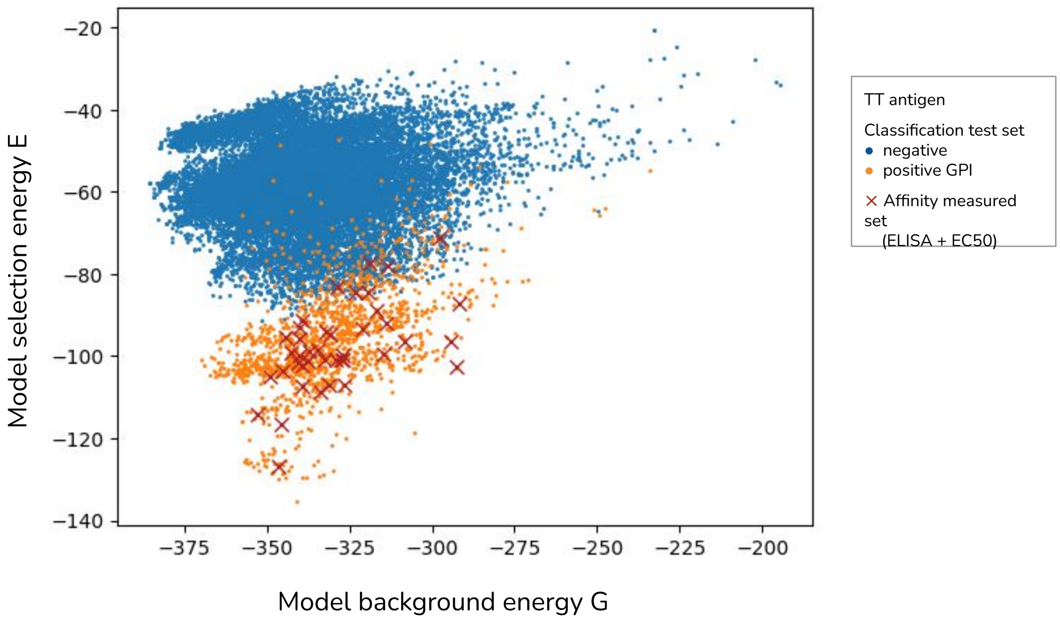

FIG. B. Antibody Repertoire Sequencing Data. Same figure of panel b of Fig 2 of the main text, but for the TT antigen: plot of background and selection energies for both negative and positive sets. Red crosses represent the validation set of antibodies with affinity measures (panel d in Fig 2 in the main text). The selection energy effectively distinguishes antibodies in the immunized repertoire from those in the unimmunized repertoire.

C. FURTHER RESULTS

Antibodies Repertoire Sequencing

In Fig B we display the scatter plot between background and selection energies inferred on tetanus toxoid immunization data.

In Fig C the performances of the standard DCA method inferred on the positive sets are shown.

Deep Mutational Scanning

In Fig D the scatter plots between the minus selective energy E and the empirical log-selectivities as defined from Eq (4) are shown for all three experiments [8, 10, 13].

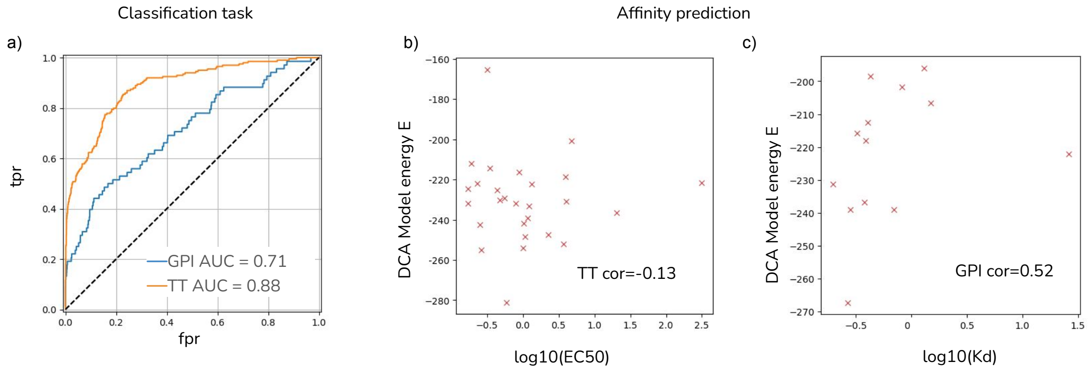

FIG. C. Alternative method: using the DCA model energy [12], trained on the positive sets. As shown in the panels it demonstrates less accuracy in both the classification of binders/non-binders and affinity prediction. Same plots as panels (c-e) in Fig 2 of the main text. (a) ROC curves for the GPI and TT cases. (b-c) DCA model energy vs. Experimental Affinity (EC50 values for TT and Kd measures for GPI).

In Fig E we report the analogous of panel (a) of Fig 3 in the main text, realized over the train datasets.

Finally, in Fig F we show an example of the capability of the AnnealDCA method to infer the initial library statistics. The scatter plots are realized from a synthetic DMS experiment, in which the initial library is generated according to a known teacher model G_{teach}, which is subsequently compared with the one inferred from the generated data, labeled as G_{stud}. The two energies turn out to be highly correlated, both on the training and test set sequences.

Experimental Evolution (EE)

In panels (c) and (d) of Fig 3 of the main text we reported respectively the sensitivity plot and the contact map related to the experiment on PSE1 of [11]. Here we show the analogous results obtained for the AAC6 and TEM-1 proteins. In panel (a) of Fig G we report the sensitivity plots for AnnealDCA, AMaLa and PlmDCA for AAC6. The first provides a positively predicted value at half-length of $\text{PPV}(L/2) = 0.51$ and an *area under the curve* (AUC) of $\text{AUC}(L/2) = 0.54$, comparable with the results provided by the AMaLa method ($\text{PPV}(L/2) = 0.51$, $\text{AUC}(L/2) = 0.51$) and significantly better than PlmDCA ($\text{PPV}(L/2) = 0.31$, $\text{AUC}(L/2) = 0.34$). In panel (b) of Fig G we show the sensitivity

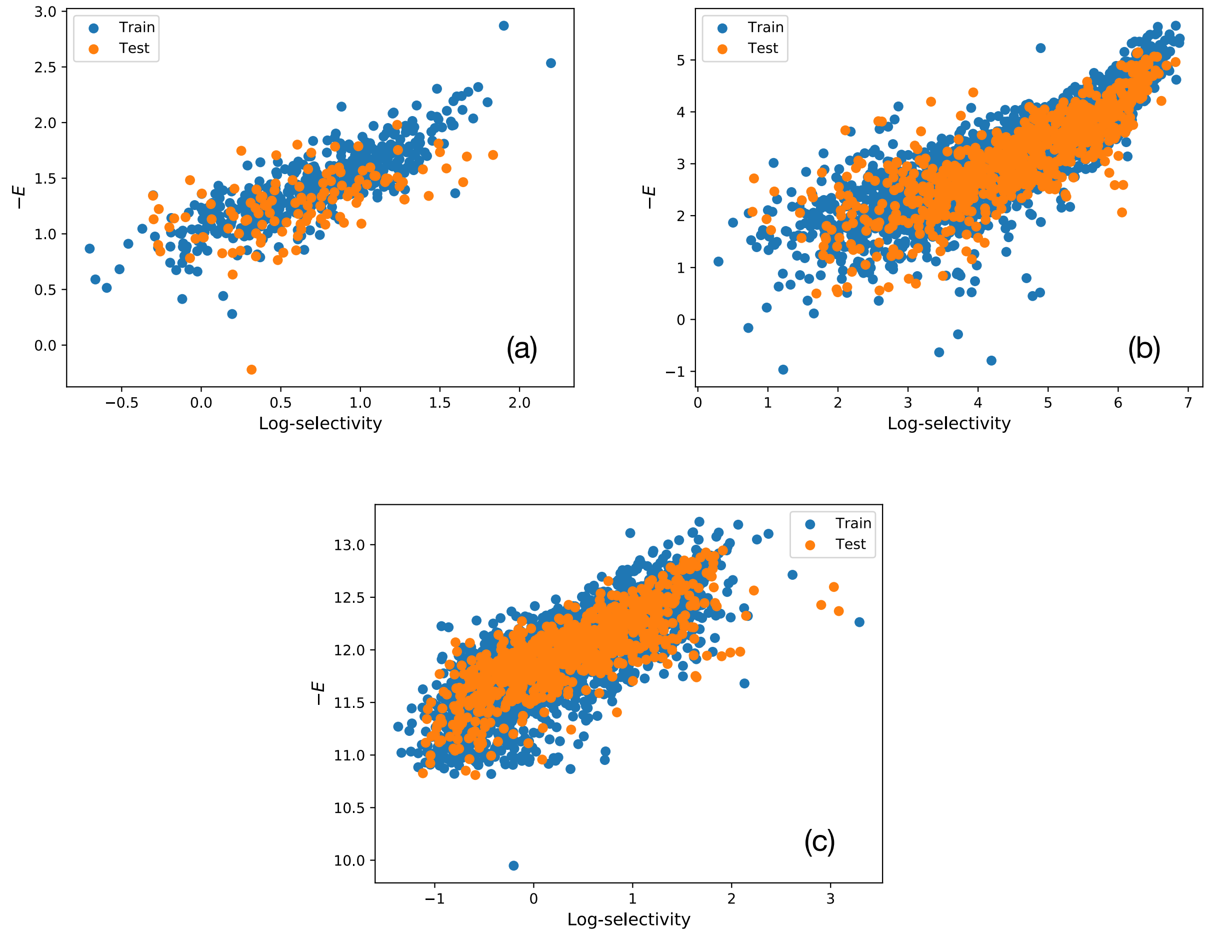

FIG. D. Scatter plots between empirical log-selectivities and minus selective energies E. All the panels are realized retaining $1/25$ of the total data points. Panel (a): scatter plot related to the dataset of [10], with Pearson correlation $\rho = 0.84$. Panel (b): scatter plot for the experiment [8], for which $\rho = 0.81$. Panel (c): scatter plot obtained from [9] data. The Pearson correlation is $\rho = 0.77$.

plots obtained for the TEM-1 protein. None of the proposed methods is able to obtain a meaningful contact prediction. In Fig H we show the contact maps obtained with the AnnealDCA and the DCA methods.

In Fig I we show a scatter plot between AnnealDCA energies G computed over a set of sequences from the AAC6 experiment [11] and their corresponding Hamming distance

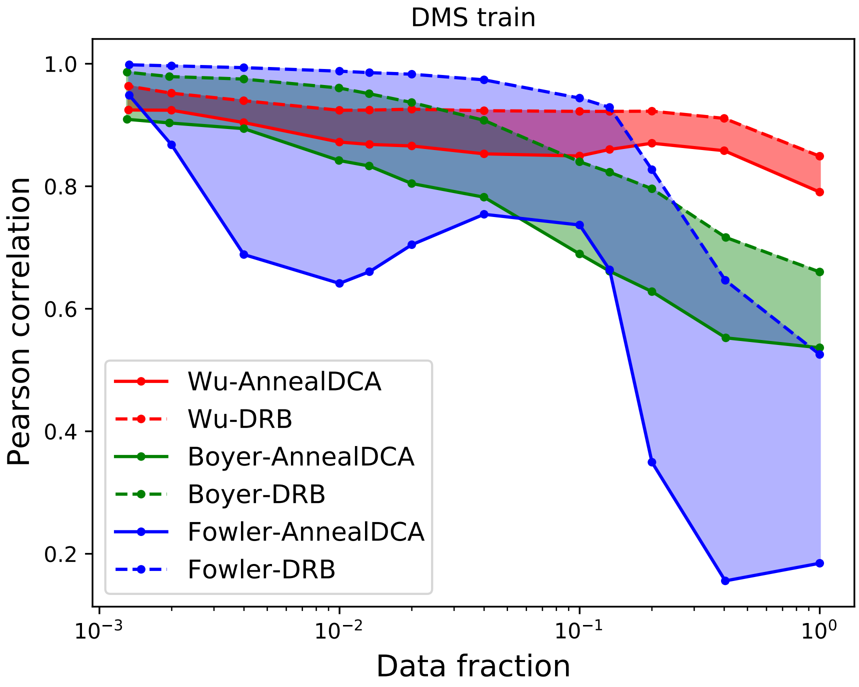

FIG. E. Comparison between AnnealDCA and DRB [14] in terms of Pearson correlation between the inferred selective energies E and the empirical log-selectivities, computed over the sequence in the train dataset. The correlation is reported as a function of the fraction of retained sequences, so to progressively exclude the ones characterized by a high uncertainty on log-selectivity.

TABLE A. Table reporting the inferred $\boldsymbol{\beta}$ values for the AnnealDCA and AMaLa methods applied to EE data.

Protein	AnnealDCA	AMaLa
PSE1	$(0, 1)$	$(1, 1.7)$
AAC6	$(0, 0.4, 1)$	$(1, 1.44, 1.89)$
TEM-1	$(0, 1, 1)$	$(0, 1, 1)$

from the wild-type sequence. The two quantities turn out to be highly correlated, as their Pearson correlation coefficient is 0.94.

Finally, we compare the values of the statistical inverse temperatures $\boldsymbol{\beta}$ inferred by both the AnnealDCA and AMaLa method in Table A. We remind that for EE data AnnealDCA takes the first sequenced round as the initial library and hence the associated inverse temperature is set to 0.

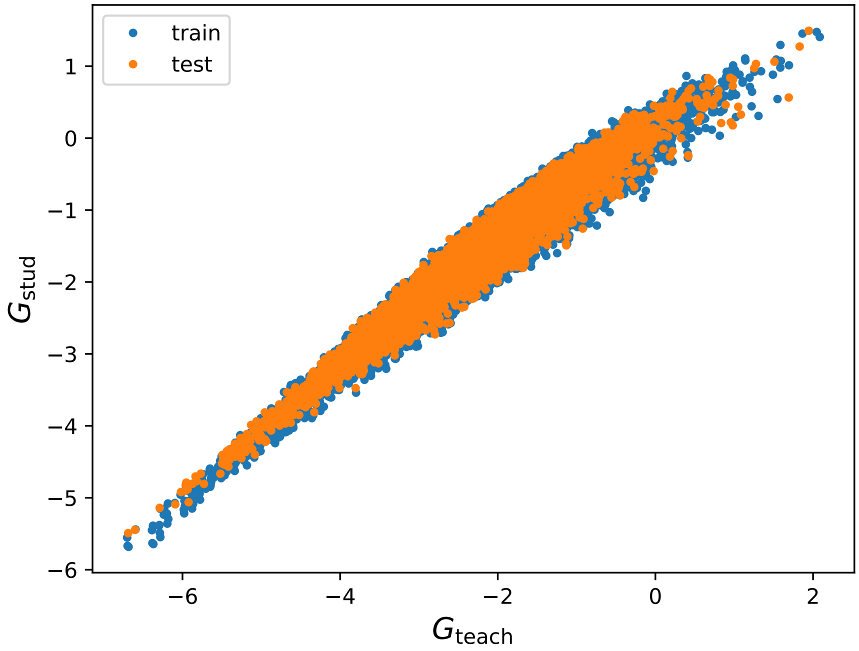

FIG. F. Scatter plots between teacher and student energies G obtained from synthetic DMS data. We simulated in-silico an analogous of Boyer et al. experiment [10]. In doing so, we generated a customized initial library, whose statistics is determined by $G_{\rm teach}$, whereas the selection process is defined by another ground-truth energy $E_{\rm teach}$. At every screening round, sequence are selected according to a survival probability $\propto \exp\left[-E_{\rm teach}(\mathbf{S})\right]$ and amplified according to a random multinomial process. Inference with AnnealDCA provides us with two estimated energetic models $G_{\rm stud}$ and $E_{\rm stud}$. The high correlation between the two initial library energies G's displayed from the scatter plots (both train and test set) shows the capability of the method to capture the statistical features of the initial library, which is fundamental to also infer an accurate selection energy E.

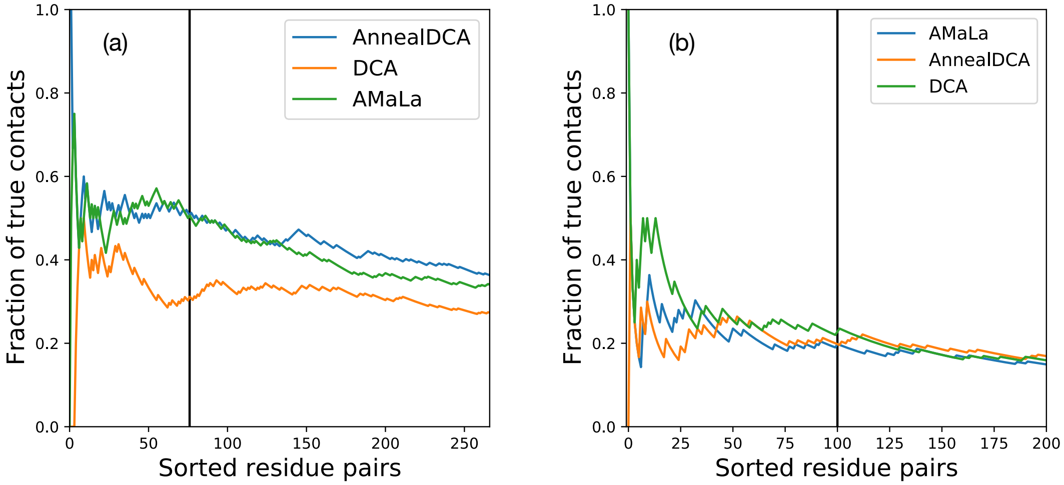

FIG. G. Sensitivity plots for AAC6 (panel (a)) and TEM-1 (panel (b)) contact prediction obtained with AnnealDCA, AMaLa and PlmDCA. On the horizontal axis the possible residue pairs are reported in a decreasing order of interaction score Eq (5). On the vertical axis the fraction of correctly predicted contacts is reported. A solid vertical line is shown in correspondence of half the protein length.

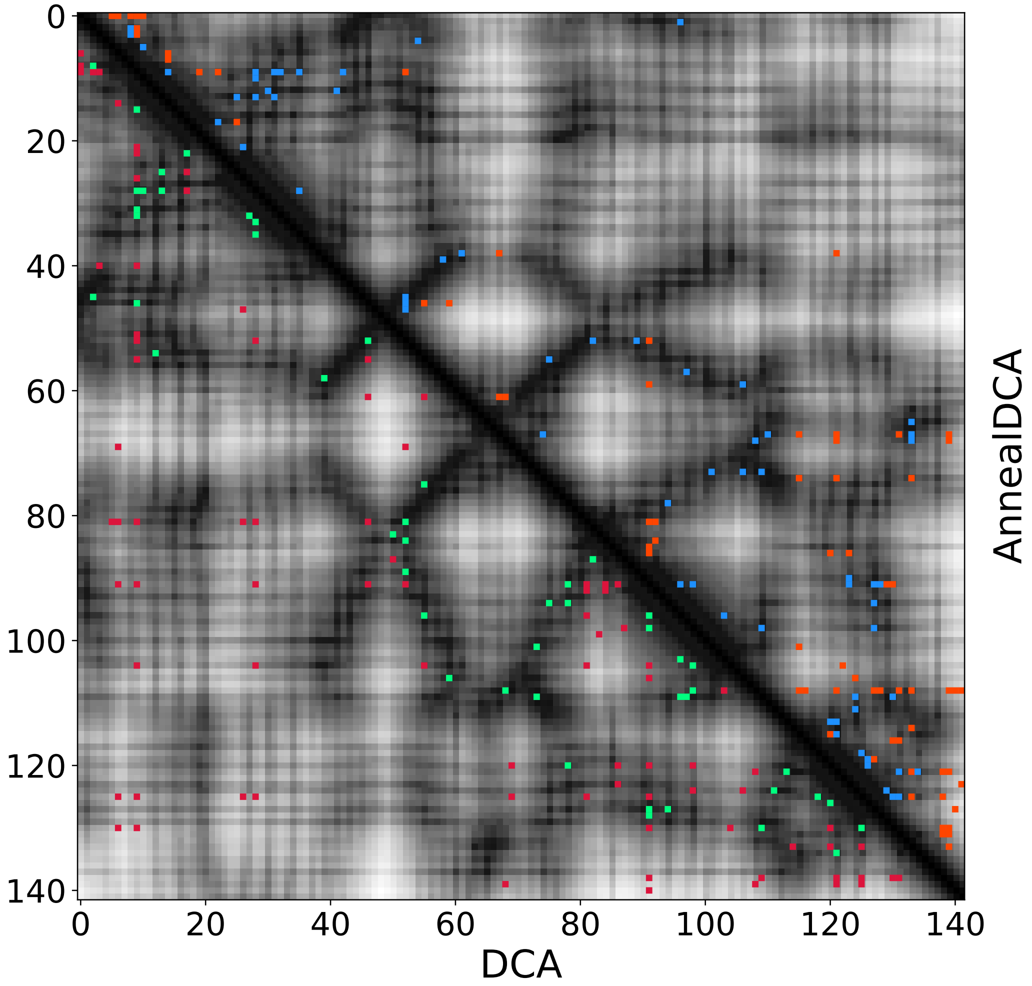

FIG. H. Contact map prediction of AAC6 protein obtained with AnnealDCA (upper-right) and PlmDCA (lower-left). Correctly predicted contacts are reported in blue for AnnealDCA and green for PlmDCA, whereas wrong predictions are reported in orange for AnnealDCA and red for PlmDCA.

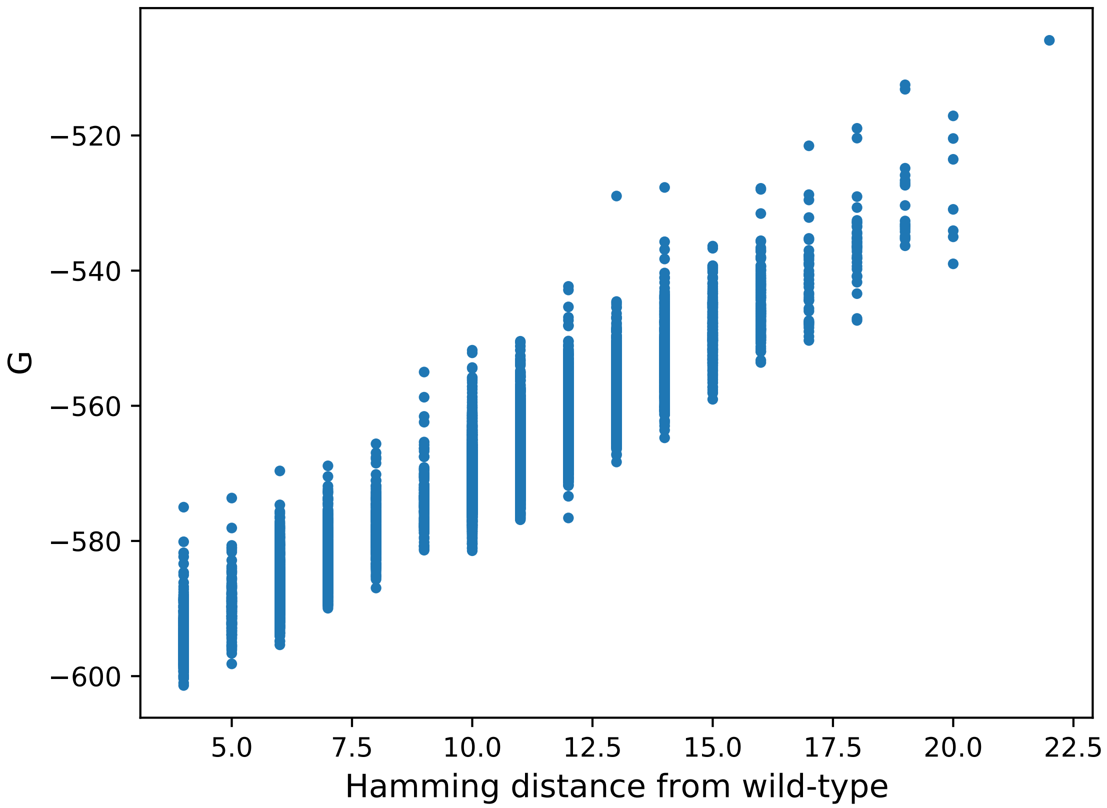

FIG. I. Scatter plot between model energies G computed over a set of sequences from AAC6 experiment and the Hamming distances of those sequences from the original wild-type.

[1] M. Ekeberg, C. Lövkvist, Y. Lan, M. Weigt, and E. Aurell, Improved contact prediction in proteins: using pseudolikelihoods to infer potts models, Physical Review E **87**, 012707 (2013).

[2] M. Ekeberg, T. Hartonen, and E. Aurell, Fast pseudolikelihood maximization for direct-coupling analysis of protein structure from many homologous amino-acid sequences, Journal of Computational Physics **276**, 341 (2014).

[3] M. Fantini, S. Lisi, P. De Los Rios, A. Cattaneo, and A. Pastore, Protein Structural Information and Evolutionary Landscape by In Vitro Evolution, Molecular Biology and Evolution **37**, 1179 (2019), https://academic.oup.com/mbe/article-pdf/37/4/1179/32960043/msz256.pdf.

[4] E. Firnberg, J. W. Labonte, J. J. Gray, and M. Ostermeier, A comprehensive, high-resolution map of a gene's fitness landscape, Molecular biology and evolution **31**, 1581 (2014).

[5] A. Gérard, A. Woolfe, G. Mottet, M. Reichen, C. Castrillon, V. Menrath, S. Ellouze, A. Poitou, R. Doineau, L. Briseno-Roa, *et al.*, High-throughput single-cell activity-based screening and sequencing of antibodies using droplet microfluidics, Nature biotechnology **38**, 715 (2020).

[6] T. A. Khan, S. Friedensohn, A. R. Gorter de Vries, J. Straszewski, H.-J. Ruscheweyh, and S. T. Reddy, Accurate and predictive antibody repertoire profiling by molecular amplification fingerprinting, Science advances **2**, e1501371 (2016).

[7] J. Dunbar and C. M. Deane, Anarci: antigen receptor numbering and receptor classification, Bioinformatics **32**, 298 (2016).

[8] N. C. Wu, L. Dai, C. A. Olson, J. O. Lloyd-Smith, and R. Sun, Adaptation in protein fitness landscapes is facilitated by indirect paths, Elife **5**, e16965 (2016).

[9] D. M. Fowler, C. L. Araya, S. J. Fleishman, E. H. Kellogg, J. J. Stephany, D. Baker, and S. Fields, High-resolution mapping of protein sequence-function relationships, Nature methods **7**, 741 (2010).

[10] S. Boyer, D. Biswas, A. Kumar Soshee, N. Scaramozzino, C. Nizak, and O. Rivoire, Hierarchy and extremes in selections from pools of randomized proteins, Proceedings of the National Academy of Sciences **113**, 3482 (2016), https://www.pnas.org/content/113/13/3482.full.pdf.

[11] M. A. Stiffler, F. J. Poelwijk, K. P. Brock, R. R. Stein, A. Riesselman, J. Teyra, S. S. Sidhu, D. S. Marks, N. P. Gauthier, and C. Sander, Protein structure from experimental evolution, Cell Systems **10**, 15 (2020).

[12] F. Morcos, A. Pagnani, B. Lunt, A. Bertolino, C. Marks, Debora S an d Sander, R. Zecchina, J. N. Onuchic, T. Hwa, and M. Weigt, Direct-coupling analysis of residue coevolution captures native contacts across many protein fami lies, Proceedings of the National Academy of Sciences **108**, E1293 (2011).

[13] D. M. Fowler and S. Fields, Deep mutational scanning: a new style of protein science, Nature methods **11**, 801 (2014).

[14] J. Fernandez-de Cossio-Diaz, G. Uguzzoni, and A. Pagnani, Unsupervised Inference of Protein Fitness Landscape from Deep Mutational Scan, Molecular Biology and Evolution 10.1093/molbev/msaa204 (2020), msaa204, https://academic.oup.com/mbe/advance-article-pdf/doi/10.1093/molbev/msaa204/33862547/msaa204.pdf.
